# Immediate and long-term outcomes after treat-all among people living with HIV in China: an interrupted time series analysis

**DOI:** 10.1186/s40249-023-01119-7

**Published:** 2023-08-14

**Authors:** Xinsheng Wu, Guohui Wu, Ping Ma, Rugang Wang, Linghua Li, Yinghui Sun, Junjie Xu, Yuwei Li, Tong Zhang, Quanmin Li, Yuecheng Yang, Lijing Wang, Xiaoli Xin, Ying Qiao, Bingxue Fang, Zhen Lu, Xinyi Zhou, Yuanyi Chen, Qi Liu, Gengfeng Fu, Hongxia Wei, Xiaojie Huang, Bin Su, Hui Wang, Huachun Zou

**Affiliations:** 1 Shenzhen Campus of Sun Yat-sen University, No. 66, Gongchang Road, Guangming District, Shenzhen, 518107 Guangdong People’s Republic of China; 2 School of Public Health (Shenzhen), Sun Yat-sen University, No. 66, Gongchang Road, Guangming District, Shenzhen, 518107 Guangdong People’s Republic of China; 3 Institute for AIDS/STD Control and Prevention, Chongqing Center for Disease Control and Prevention, Chongqing, People’s Republic of China; 4 Department of Infectious Diseases, Tianjin Second People’s Hospital, Tianjin, People’s Republic of China; 5 Tianjin Association of STD/AIDS Prevention and Control, Tianjin, People’s Republic of China; 6 Dalian Public Health Clinical Center, Dalian, People’s Republic of China; 7 Infectious Disease Center, Guangzhou Eighth People’s Hospital, Guangzhou Medical University, Guangzhou, People’s Republic of China; 8 Clinical Research Academy, Peking University Shenzhen Hospital, Peking University, Shenzhen, People’s Republic of China; 9 Clinical and Research Center for Infectious Diseases, Beijing Youan Hospital, Capital Medical University, No.8 Xitoutiao, Youanmenwai, Feng Tai District, Beijing, 100069 People’s Republic of China; 10 Dehong Prefecture Center for Disease Control and Prevention, Dehong, People’s Republic of China; 11 Shijiazhuang Fifth Hospital, Shijiazhuang, People’s Republic of China; 12 No.6 People’s Hospital of Shenyang, Shenyang, People’s Republic of China; 13 No.2 Hospital of Hohhot, Hohhot, People’s Republic of China; 14 Department of STD/AIDS Control and Prevention, Jiangsu Provincial Center for Disease Control and Prevention, Nanjing, People’s Republic of China; 15 Department of Infectious Disease, The Second Hospital of Nanjing, Nanjing University of Chinese Medicine, 1-1 Zhongfu Road, Nanjing, 210036 Jiangsu People’s Republic of China; 16 National Clinical Research Centre for Infectious Diseases, The Third People’s Hospital of Shenzhen and The Second Affiliated Hospital of Southern, University of Science and Technology, Bulan Road 29#, Longgang District, Shenzhen, 518112 Guangdong People’s Republic of China; 17 School of Public Health, Fudan University, 130 Dongan Road, Xuhui District, Shanghai, 200032 People’s Republic of China; 18 School of Public Health, Southwest Medical University, Luzhou, People’s Republic of China; 19 Kirby Institute, University of New South Wales, Sydney, Australia

**Keywords:** HIV, Antiretroviral therapy, Treat-all, CD4

## Abstract

**Background:**

In 2003, China implemented free antiretroviral therapy (ART) for people living with HIV (PLHIV), establishing an eligibility threshold of CD4 < 200 cells/μl. Subsequently, the entry criteria were revised in 2012 (eligibility threshold: CD4 ≤ 350 cells/μl), 2014 (CD4 ≤ 500 cells/μl), and 2016 (treat-all). However, the impact of treat-all policy on HIV care and treatment indicators in China is unknown. We aimed to elucidate the immediate and long-term impact of the implementation of treat-all policy in China.

**Methods:**

Anonymized programmatic data on ART initiation and collection in PLHIV who newly started ART were retrieved between 1 January 2015 and 31 December 2019, from two provincial and municipal Centers for Disease Control and Prevention and ten major infectious disease hospitals specialized in HIV care in China. We used Poisson and quasi-Poisson segmented regression models to estimate the immediate and long-term impact of treat-all on three key indicators: monthly proportion of 30-day ART initiation, mean CD4 counts (cells/μl) at ART initiation, and mean estimated time from infection to diagnosis (year). We built separate models according to gender, age, route of transmission and region.

**Results:**

Monthly data on ART initiation and collection were available for 75,516 individuals [gender: 83.8% males; age: median 39 years, interquartile range (IQR): 28–53; region: 18.5% Northern China, 10.9% Northeastern China, 17.5% Southern China, 49.2% Southwestern China]. In the first month of treat-all, compared with the contemporaneous counterfactual, there was a significant increase in proportion of 30-day ART initiation [+ 12.6%, incidence rate ratio (IRR) = 1.126, 95% *CI*: 1.033–1.229; *P* = 0.007] and mean estimated time from infection to diagnosis (+ 7.0%, IRR = 1.070, 95% *CI*: 1.021–1.120; *P* = 0.004), while there was no significant change in mean CD4 at ART initiation (IRR = 0.990, 95% *CI*: 0.956–1.026; *P* = 0.585). By December 2019, the three outcomes were not significantly different from expected levels. In the stratified analysis, compared with the contemporaneous counterfactual, mean CD4 at ART initiation showed significant increases in Northern China (+ 3.3%, IRR = 1.033, 95% *CI*: 1.001–1.065; *P* = 0.041) and Northeastern China (+ 8.0%, IRR = 1.080, 95% *CI*: 1.003–1.164; *P* = 0.042) in the first month of treat-all; mean estimated time from infection to diagnosis showed significant increases in male (+ 5.6%, IRR = 1.056, 95% *CI*: 1.010–1.104; *P* = 0.016), female (+ 14.8%, IRR = 1.148, 95% *CI*: 1.062–1.240; *P* < 0.001), aged 26–35 (+ 5.3%, IRR = 1.053, 95% *CI*: 1.001–1.109; *P* = 0.048) and > 50 (+ 7.8%, IRR = 1.078, 95% *CI*: 1.000–1.161; *P* = 0.046), heterosexual transmission (+ 12.4%, IRR = 1.124, 95% *CI*: 1.042–1.213; *P* = 0.002) and Southwestern China (+ 12.9%, IRR = 1.129, 95% *CI*: 1.055–1.208; *P* < 0.001) in the first month of treat-all.

**Conclusions:**

The implementation of treat-all policy in China was associated with a positive effect on HIV care and treatment outcomes. To advance the work of rapid ART, efforts should be made to streamline the testing and ART initiation process, provide comprehensive support services, and address the issue of uneven distribution of medical resources.

**Supplementary Information:**

The online version contains supplementary material available at 10.1186/s40249-023-01119-7.

## Background

Since the introduction of antiretroviral therapy (ART) as the standard treatment for people living with HIV (PLHIV), numerous studies have consistently demonstrated that initiating ART early leads to longer and healthier lives for PLHIV [1–4]. Additionally, increased treatment coverage helps protect the general population by reducing the risk of infection[5]. Recognizing these benefits, the World Health Organization has consistently advocated for earlier ART initiation and recommended the implementation of treat-all (or universal test and treat, UTT) policy in 2016. This policy removes all restrictions on ART eligibility for PLHIV [6]. Real-world data provided evidence supporting the advantages of this policy in practice, including increased mean CD4 counts at ART initiation [7], reduced time from diagnosis to ART initiation [8, 9], improved retention rates [10, 11], and declined attrition (loss to follow-up at 12 months post-HIV diagnosis) [12].

Although treat-all guidelines have been adopted by almost all countries [13], only a few studies have been conducted outside of Africa. China implemented free ART for PLHIV in 2003, and gradually increased the eligibility threshold from 200 cells/µl in 2003 to 350 cells/µl in 2012 and 500 cells/µl in 2014 to encourage early ART initiation. The number of facilities engaged in ART increased from 671 in 2004 to 3733 in 2013, and the number of ART initiation in the same year increased from 9562 in 2004 to 70,360 in 2013 [15]. In June 2016, China implemented a treat-all policy, which removed any threshold for CD4 + T-cell count and recommended immediate ART initiation following diagnosis for all PLHIV on a voluntary basis. Moreover, HIV prevention and control agencies in all regions were required to enhance testing efforts to identify as many HIV-infected individuals as possible. A study conducted in Yunnan, Southwest China [16] found that among 4,409 newly reported cases of heterosexual HIV infection in 2016–2018, the rates of ART initiation within 7 days increased steadily over this period, from 38.82% in 2016 to 41.46% in 2017, and 47.99% in 2018. Another study focused on men who have sex with men (MSM) in Chengdu, Southwest China [17], revealed that ART coverage rate increased from 11.11% in 2008 to 92.29% in 2018, with an average annual percent change of 16.09% (95% *CI*: 11.76%–20.59%). However, it is important to note that these results have limited generalizability, and the impact of the treat-all policy in the broader context of China remains unclear.

To assess the impact of the treat-all policy, we analyzed the time from diagnosis to ART initiation, the CD4 + T-cell count at ART initiation, and the mean estimated time from infection to diagnosis. The CD4 + T-cell count at ART initiation serves as an indicator of the immune status and the risk of opportunistic infections at the start of treatment for PLHIV. Understanding the change in time from diagnosis to ART initiation and CD4 + T-cell count at ART initiation before and after treat-all may help shed light on the effectiveness of this policy and offer insights for future policy development and implementation. To analyse changes in universal test efforts, we employed the back-calculation method to determine the average of the estimated time from infection to diagnosis for patients initiating ART, based on the CD4 elimination model parameters estimated from HIV/AIDS data in the National AIDS Case Report Database [18, 19]. Through long-term observation of a large population covering various regions in China, we conducted an interrupted time series analysis by fitting a Poisson or quasi-Poisson segmented regression model with Newey-West standard errors to elucidate the immediate and long-term impact of the implementation of the treat-all policy.

## Methods

### Study design

We performed an interrupted time series analysis using data retrieved from the National Free Antiretroviral Treatment Program (NFATP) database [20]. In China, the NFATP was launched in 2002, and is managed by the National Center for AIDS/STD Control and Prevention, Chinese Center for Disease Control and Prevention (China CDC). Further details on the description and collection of this database have been provided in similar studies [21]. Anonymized programmatic data on ART initiation and collection in PLHIV who newly started ART between January 1, 2015 and December 31, 2019 were collected from two provincial and municipal CDCs in Chongqing and Dehong, and ten major infectious disease hospitals specialized in HIV care in Guangzhou, Shenzhen, Nanjing, Hohhot, Tianjin, Shenyang, Beijing, Shijiazhuang and Dalian in China. Baseline and follow-up information of PLHIV was obtained, including gender, date of birth, route of transmission, time of HIV diagnosis, time of ART initiation, time of follow-up, CD4 + T-cell count, among others.

In China, CDCs at various levels have been established to implement public health technical management and services, with a specific responsibility for HIV surveillance. PLHIV are required to initiate ART and pick up drugs at designated infectious disease hospitals. Chongqing is a municipality and Dehong is an autonomous minority prefecture in Southwestern China. Guangzhou and Shenzhen are located in Southern China; Hohhot, Beijing, Tianjin and Shijiazhuang are cities in Northern China; Shenyang and Dalian are located in Northeastern China; Nanjing is a municipality in Eastern China. These regions collectively represent 20.8% of newly reported HIV/AIDS cases in China in 2019, and encompass both urban and rural areas with diverse socio-economic conditions across China.

### Outcomes

The primary outcomes of interest were the monthly proportions of 30-day ART initiation (from diagnosis, 100%), mean CD4 + T-cell count at ART initiation (CD4 count, cells/μl), and mean estimated time from infection to diagnosis (mean estimated time to diagnosis, year) before and after the implementation of treat-all (June 2016). These three indicators were selected based on their importance in monitoring HIV care and treatment outcomes and aligning with the goals of the treat-all policy. Proportion of 30-day ART initiation refers to the proportion of ART initiation within 30 days of diagnosis, which measures how quickly eligible patients were initiated on ART after diagnosis. According to the recorded dates of HIV-positive diagnosis and initiating ART of PLHIV in the database, we calculated the time interval for each patient. We then calculated the proportion of PLHIV who initiated ART within 30 days among those who started ART per month. Mean CD4 count refers to the mean CD4 + T cell counts at ART initiation, which provides insight into the immune status of patients at the time of starting treatment. Estimated time to diagnosis refers to the mean estimated year from infection to diagnosis, calculated by back-calculation method based on CD4 elimination model fitted using HIV/AIDS data in the National AIDS Case Report Database in China [18, 19, 22] (Additional file 1: Appendix).

We stratified outcome data by gender, age, route of transmission and region. Age was divided into 0–25, 26–35, 36–50 and > 50 years groups. Route of transmission was divided into heterosexual, homosexual and others (including blood transfusion, intravenous drug use, mother-to-child transmission, etc.). Region was divided into Northern China (Hohhot, Shijiazhuang, Beijing and Tianjin), Northeastern China (Dalian and Shenyang), Southern China (Guangzhou and Shenzhen), Southwestern China (Chongqing and Dehong), and Eastern China (Nanjing).

### Statistical analysis

We used descriptive statistics to summarize data on outcomes before and after treat-all. The median and interquartile range (IQR) were used to describe continuous variables, and frequencies and proportions were used to describe categorical variables. Characteristics of PLHIV before and after treat-all were compared using *χ*^2^ tests for categorical variables and Kruskal–Wallis test for continuous variables.

As a common method for studying changes in HIV-related indicators [10, 23–25], interrupted time series analysis was used to assess whether exposure to the implementation of treat-all affected these outcomes. We performed Poisson or quasi-Poisson segmented regression models to estimate the immediate impact of treat-all on these outcomes, as well as their trends. Quasi-Poisson model was used to capture overdispersion that occurs in the proportions of 30-day ART initiation. The model for each outcome included a time variable, a dummy variable indicating the implementation of treat-all policy, and an interaction term between the time and dummy variables (Additional file 1: Appendix). To adjust the long-term trends, we used models before treat-all to predict data after June 2016 without treat-all, which represents the counterfactual scenario.

Incidence rate ratio (IRR) was calculated by comparing the fitted numbers from the model with the expected numbers from the contemporaneous counterfactual. By subtracting a time period from the time variable, we were able to center time at the June 2016 (treat-all) and December 2019 in order to estimate the immediate and long-term impact. Expected level was defined as the expected numbers from the counterfactual model in December 2019 [23–25]. To calculate the trend (absolute) after treat-all, we added together the coefficients associated with time and the time-dummy interaction. To eliminate autocorrelation and heteroskedasticity, we adjusted the standard errors of the parameters of the model by the Newey-west method to calculate the 95% confidence interval (CI) for the IRR, with the lag taking the optimal value calculated through the automatic bandwidth selection procedure [26]. We built separate models according to gender, age, route of transmission and region. To account for potential seasonal changes in clinic activity of the three outcomes, we did a sensitivity analysis with two pairs of sine and cosine terms (Fourier terms) included in the model [27].

All statistical tests were two sided, and *P* < 0.05 was considered statistically significant. All statistical analyses were conducted using R 4.2.1 (R Foundation for Statistical Computing, Vienna, Austria; Appendix).

## Results

### Overview of study

A total of 75,516 individuals on ART initiation and collection were recorded from January 2015 to December 2019. There were 19,938 in the 17 months before treat-all (January 2015 to May 2016) and 55,578 in the 43 months after treat-all (June 2016 to December 2019), respectively. Overall, males accounted for 83.8% of the total sample, and the median age was 39 years [interquartile range (IQR): 28–53]. Detailed characteristics of individuals included were provided in Table 1.

**Table 1:** Demographics and HIV diagnosis and ART initiation among PLHIV in China 1 January 2015 to 31 December 2019

	Total	Before treat-all	After treat-all	*P* value
Total number in study period	75,516	19,938	55,578	
Gender (%)				< 0.001
Male	63,279 (83.8%)	16,970 (85.1%)	46,309 (83.3%)	
Female	12,237 (16.2%)	2968 (14.9%)	9269 (16.7%)	
Age, years (%)				< 0.001
Median (IQR)	39 (28–53)	36 (27–49)	40 (28–54)	
0–25	13,525 (17.9%)	4038 (20.3%)	9487 (17.1%)	
26–35	21,959 (29.1%)	6468 (32.4%)	15,491 (27.9%)	
36–50	19,961 (26.4%)	5526 (27.7%)	14,435 (26.0%)	
> 50	20,071 (26.6%)	3906 (19.6%)	16,165 (29.1%)	
Region (%)				< 0.001
Northern China	14,005 (18.5%)	4170 (20.9%)	9835 (17.7%)	
Northeastern China	8238 (10.9%)	2197 (11.0%)	6041 (10.9%)	
Southern China	13,238 (17.5%)	3862 (19.4%)	9376 (16.9%)	
Southwestern China	37,163 (49.2%)	8793 (44.1%)	28,370 (51.0%)	
Route of transmission (%)				< 0.001
Heterosexual	32,030 (42.4%)	7125 (35.7%)	24,905 (44.8%)	
Homosexual	27,227 (36.1%)	8050 (40.4%)	19,177 (34.5%)	
Others	16,259 (21.5%)	4763 (23.9%)	11,496 (20.7%)	
Estimated time from infection to diagnosis (year)				< 0.001
Median (IQR)	5.9 (3.2–9.4)	5.3 (3.0–8.2)	6.1 (3.3–9.9)	
Time from diagnosis to ART initiation (day)				< 0.001
Median (IQR)	26 (12–98)	41 (18–256)	22 (11–65)	
CD4 at ART initiation (cells/μl)				< 0.001
Median (IQR)	247 (114–376)	277 (158–389)	235 (98–370)	

*ART* Antiretroviral therapy, *PLHIV* People living with HIV, *IQR* Interquartile range. Before treat-all: 1 January 2015–31 May 2016. After treat-all: 1 June 2016–31 December 2019. Region was divided into Northern China (Hohhot, Shijiazhuang, Beijing and Tianjin), Northeastern China (Dalian and Shenyang), Southern China (Guangzhou and Shenzhen), Southwestern China (Chongqing and Dehong), and Eastern China (Nanjing)

### Proportions of 30-day ART initiation

**Fig. 1:**
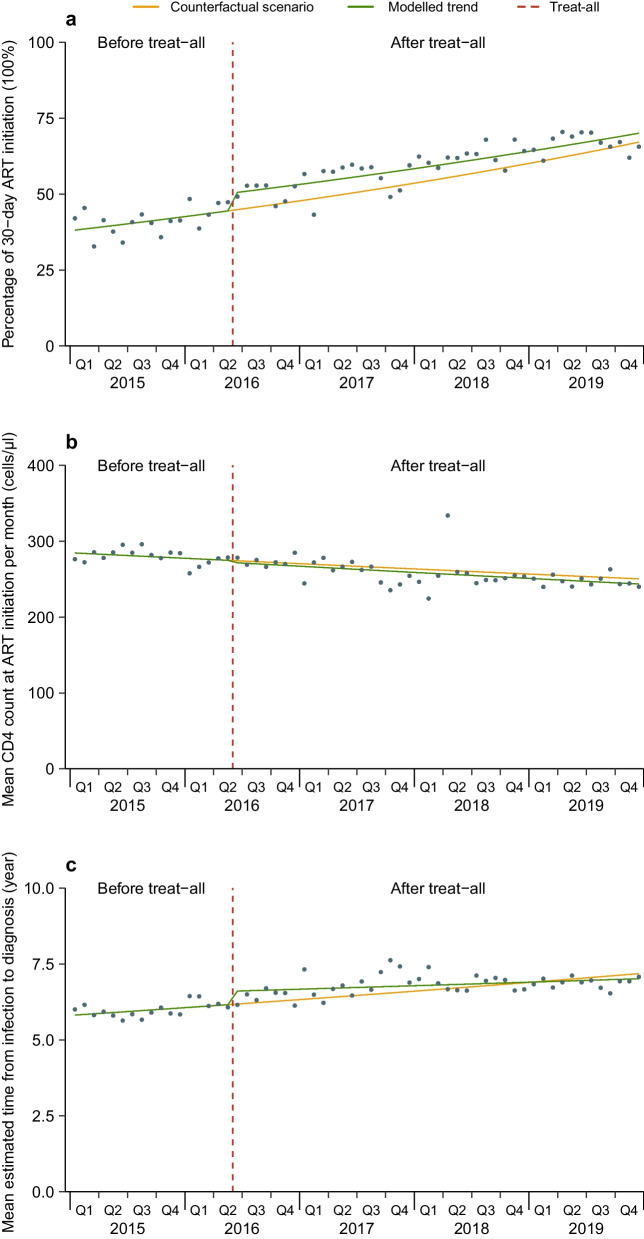
Monthly numbers, trends and fitted Poisson or quasi-Poisson segmented regression models for three outcomes in China from 1 January 2015 to 31 December 2019, not accounting for seasonality^**#**^. Percentage of 30 − day ART initiation (**a**), mean CD4 count at ART initiation (**b**) and mean estimated time from infection to diagnosis (**c**). #: three indicators include proportion of 30-day ART initiation, mean CD4 counts (cells/μl) at ART initiation, and mean estimated time from infection to diagnosis (year). ART: Antiretroviral therapy. treat-all: June 2016. Percentage of 30-day ART initiation was modelled using quasi-Poisson segmented regression model. Mean CD4 count at ART initiation and mean estimated time from infection to diagnosis was modelled using Poisson segmented regression model

**Fig. 2:**
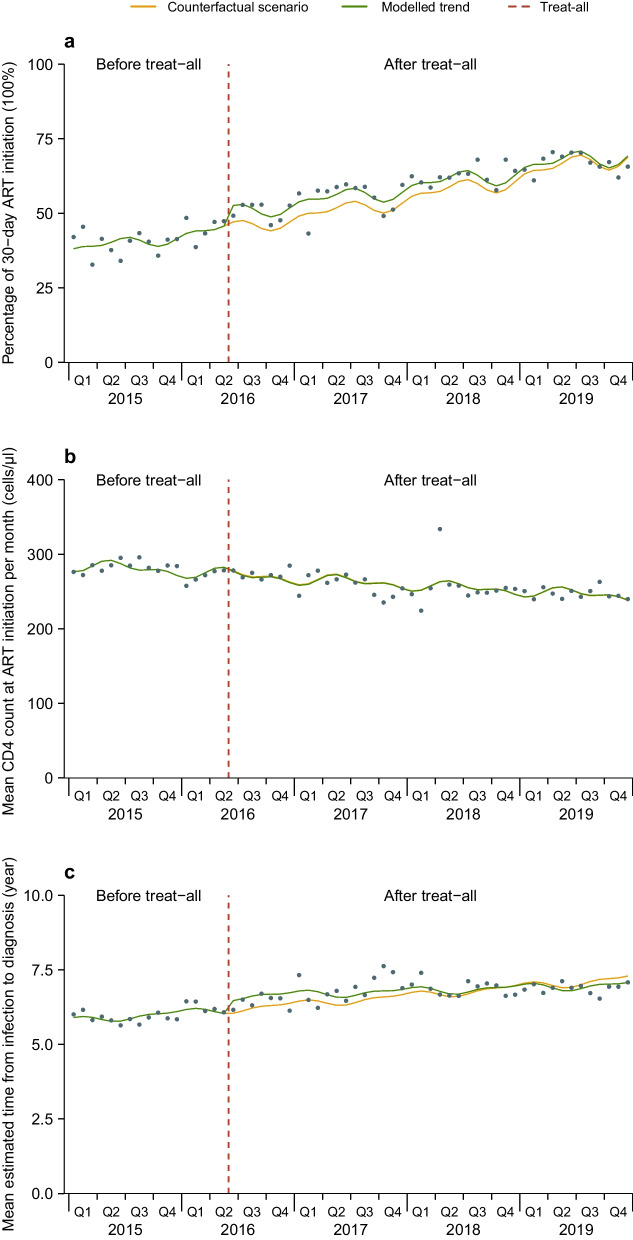
Monthly numbers, trends and fitted Poisson or quasi-Poisson segmented regression models for three outcomes in China from 1 January 2015 to 31 December 2019, accounting for seasonality^**#**^. Percentage of 30 − day ART initiation (**a**), mean CD4 count at ART initiation (**b**) and mean estimated time from infection to diagnosis (**c**). #: three indicators include proportion of 30-day ART initiation, mean CD4 counts (cells/μl) at ART initiation, and mean estimated time from infection to diagnosis (year). ART: Antiretroviral therapy. treat-all: June 2016. Percentage of 30 − day ART initiation was modelled using quasi-Poisson segmented regression model. Mean CD4 count at ART initiation and mean estimated time from infection to diagnosis was modelled using Poisson segmented regression model

The median proportions of 30-day ART initiation in each month recorded before and after treat-all were 41.4% (IQR: 38.7–43.3) and 60.3% (IQR: 56.0–65.1), respectively. Quasi-Poisson segmented regression analysis showed that compared with the contemporaneous counterfactual, the proportion of 30-day ART initiation increased by 12.6% in the first month of treat-all (June 2016) [incidence rate ratio (IRR): 1.126; 95% confidence interval (*CI*): 1.033–1.229; *P* = 0.007; Table 2 and Fig. 1A], but not significant in subgroups; proportion of 30-day ART initiation showed an increase trend of 1.0% per month before treat-all (IRR = 1.010, 95% *CI*: 1.001–1.018; *P* = 0.030), and after treat-all showed an increasing trend of 0.8% per month (IRR = 1.008, 95% *CI*: 1.006–1.010; *P* < 0.001); by the end of 2019 (December 2019), proportion of 30-day ART initiation was not significantly different from the expected level (IRR = 1.044, 95% *CI*: 0.681–1.600; *P* = 0.844). In the stratified analysis, proportion of 30-day ART initiation was significantly lower than expected level by the end of 2019 for male (IRR = 0.484, 95% *CI*: 0.256–0.916; *P* = 0.026), aged 0–25 (IRR = 0.400, 95% *CI*: 0.202–0.790; *P* = 0.008) and 26–35 (IRR = 0.425, 95% *CI*: 0.248–0.728; *P* = 0.002), homosexual transmission (IRR = 0.469, 95% *CI*: 0.238–0.926; *P* = 0.029) and Northeastern China (IRR = 0.423, 95% *CI*: 0.247–0.722; *P* = 0.002). The results were similar in a sensitivity analysis considering seasonality (Table 5 and Fig. 2A).

**Table 2:** Quasi-Poisson segmented regression models of the impact of treat-all on proportion of 30-day ART initiation among PLHIV in China 1 January 2015 to 31 December 2019

	IRR (95% *CI*)							
	Level change at treat-all	*P* value	Level change in December 2019	*P* value	Trend before treat-all	*P* value	Trend after treat-all	*P* value
Proportion of 30-day ART initiation	1.126 (1.033–1.229)	0.007	1.044 (0.681–1.600)	0.844	1.010 (1.001–1.018)	0.030	1.008 (1.006–1.010)	< 0.001
Gender								
Male	0.969 (0.859–1.095)	0.617	0.484 (0.256–0.916)	0.026	1.015 (1.002–1.029)	0.031	0.999 (0.998–1.000)	0.068
Female	1.048 (0.831–1.321)	0.692	1.003 (0.284–3.540)	0.997	1.006 (0.981–1.032)	0.638	1.005 (1.002–1.008)	0.002
Age, years								
0–25	0.968 (0.833–1.125)	0.671	0.400 (0.202–0.790)	0.008	1.013 (0.999–1.028)	0.091	0.992 (0.991–0.994)	< 0.001
26–35	0.941 (0.845–1.048)	0.268	0.425 (0.248–0.728)	0.002	1.014 (1.003–1.025)	0.016	0.995 (0.993–0.996)	< 0.001
36–50	0.967 (0.832–1.123)	0.658	0.599 (0.237–1.513)	0.279	1.010 (0.991–1.030)	0.292	0.999 (0.997–1.001)	0.246
> 50	1.100 (0.871–1.388)	0.423	0.712 (0.184–2.762)	0.623	1.021 (0.992–1.050)	0.153	1.010 (1.007–1.013)	< 0.001
Route of transmission								
Heterosexual	1.076 (0.877–1.320)	0.482	0.769 (0.209–2.833)	0.693	1.014 (0.987–1.042)	0.316	1.006 (1.003–1.009)	< 0.001
Homosexual	0.949 (0.826–1.091)	0.462	0.469 (0.238–0.926)	0.029	1.012 (0.998–1.026)	0.101	0.995 (0.992–0.997)	< 0.001
Others	0.907 (0.721–1.140)	0.401	0.345 (0.090–1.327)	0.122	1.018 (0.990–1.047)	0.210	0.995 (0.992–0.998)	0.001
Region								
Northern China	0.875 (0.681–1.122)	0.293	0.371 (0.117–1.178)	0.093	1.017 (0.993–1.041)	0.163	0.996 (0.993–1.000)	0.058
Northeastern China	1.146 (0.966–1.359)	0.118	0.423 (0.247–0.722)	0.002	1.015 (1.004–1.026)	0.007	0.991 (0.987–0.995)	< 0.001
Southern China	0.923 (0.725–1.175)	0.516	0.278 (0.064–1.209)	0.088	1.022 (0.991–1.053)	0.171	0.993 (0.990–0.996)	< 0.001
Southwestern China	1.050 (0.856–1.289)	0.637	0.875 (0.241–3.176)	0.839	1.010 (0.983–1.037)	0.493	1.005 (1.003–1.008)	< 0.001
Eastern China	0.757 (0.632–1.006)	0.060	0.498 (0.264–1.040)	0.061	1.011 (0.998–1.024)	0.108	1.001 (0.998–1.004)	0.672

*IRR* Incidence rate ratio, *CI* Confidence interval, *ART* Antiretroviral therapy. Treat-all: June 2016. PLHIV: People living with HIV. Region was divided into Northern China (Hohhot, Shijiazhuang, Beijing and Tianjin), Northeastern China (Dalian and Shenyang), Southern China (Guangzhou and Shenzhen), Southwestern China (Chongqing and Dehong), and Eastern China (Nanjing)

### CD4 count at ART initiation

The median CD4 at initiation recorded before treat-all was 277 cells/μl (IQR: 158–389), while after treat-all was 235 cells/μl (IQR: 98–370). Poisson segmented regression analysis showed no significant change in mean CD4 in the first month of treat-all (0.990, 0.956–1.026; *P* = 0.585; Table 3 and Fig. 1B). Before treat-all mean CD4 showed a not significant trend per month (IRR = 0.998, 95% *CI*: 0.995–1.001; *P* = 0.196), after treat-all showed a decreasing trend of 0.3% per month (IRR = 0.997, 95% *CI*: 0.997–0.998; *P* < 0.001); by the end of 2019, mean CD4 was not significantly different from expected level (IRR = 0.973, 95% *CI*: 0.842–1.124; *P* = 0.709). In the stratified analysis, mean CD4 in females (IRR = 0.932, 95% *CI*: 0.891–0.976; *P* = 0.003) and Southwestern China (IRR = 0.926, 95% *CI*: 0.867–0.989; *P* = 0.022) showed significant decreases in the first month of treat-all, while it showed significant increases in Northern China (IRR = 1.033, 95% *CI*: 1.001–1.065; *P* = 0.041) and Northeastern China (IRR = 1.080, 95% *CI*: 1.003–1.164; *P* = 0.042). By the end of 2019, mean CD4 in females (IRR = 1.124, 95% *CI*: 1.092–1.156; *P* < 0.001) and Eastern China (IRR = 2.396, 95% *CI*: 1.589–3.614; *P* < 0.001) was higher than expected levels. The results were similar across age and route of transmission groups, and in a sensitivity analysis considering seasonality (Table 5 and Fig. 2B).

**Table 3:** Poisson segmented regression models of the impact of treat-all on mean CD4 at ART initiation among PLHIV in China 1 January 2015 to 31 December 2019

	IRR (95% *CI*)							
	Level change at treat-all	*P* value	Level change in December 2019	*P* value	Trend before treat-all	*P* value	Trend after treat-all	*P* value
Mean CD4	0.990 (0.956–1.026)	0.585	0.973 (0.842–1.124)	0.709	0.998 (0.995–1.001)	0.196	0.997 (0.997–0.998)	< 0.001
Gender								
Male	1.001 (0.968–1.036)	0.941	0.955 (0.822–1.109)	0.548	0.998 (0.995–1.001)	0.213	0.997 (0.996–0.998)	< 0.001
Female	0.932 (0.891–0.976)	0.003	1.124 (1.092–1.156)	< 0.001	0.996 (0.995–0.996)	< 0.001	1.000 (0.999–1.001)	0.567
Age, years								
0–25	1.015 (0.976–1.056)	0.447	0.932 (0.748–1.160)	0.526	1.001 (0.997–1.006)	0.553	0.999 (0.999–1.000)	0.081
26–35	1.002 (0.967–1.038)	0.926	0.881 (0.751–1.033)	0.118	1.001 (0.998–1.004)	0.553	0.998 (0.997–0.999)	< 0.001
36–50	1.007 (0.944–1.074)	0.841	1.134 (0.915–1.404)	0.250	0.995 (0.991–0.999)	0.022	0.998 (0.997–1.000)	0.060
> 50	0.971 (0.876–1.076)	0.572	1.340 (0.921–1.949)	0.126	0.992 (0.985–0.998)	0.014	0.999 (0.998–1.000)	0.137
Route of transmission								
Heterosexual	0.936 (0.852–1.027)	0.163	1.137 (0.866–1.493)	0.357	0.995 (0.989–1.000)	0.058	0.999 (0.998–1.000)	0.170
Homosexual	1.038 (0.994–1.084)	0.093	0.941 (0.837–1.057)	0.304	1.000 (0.997–1.002)	0.743	0.997 (0.996–0.998)	< 0.001
Others	1.024 (0.971–1.080)	0.380	0.908 (0.704–1.171)	0.458	1.001 (0.996–1.006)	0.727	0.998 (0.997–0.999)	0.001
Region								
Northern China	1.033 (1.001–1.065)	0.041	0.916 (0.808–1.040)	0.175	1.002 (1.000–1.004)	0.042	0.999 (0.998–1.000)	0.113
Northeastern China	1.080 (1.003–1.164)	0.042	0.805 (0.689–0.941)	0.007	1.002 (0.999–1.005)	0.126	0.995 (0.993–0.997)	< 0.001
Southern China	1.004 (0.939–1.074)	0.900	0.700 (0.475–1.031)	0.071	1.006 (0.998–1.014)	0.118	0.998 (0.996–0.999)	0.001
Southwestern China	0.926 (0.867–0.989)	0.022	1.145 (0.875–1.499)	0.322	0.993 (0.988–0.998)	0.010	0.998 (0.997–0.999)	0.003
Eastern China	1.109 (0.971–1.268)	0.128	2.396 (1.589–3.614)	< 0.001	0.982 (0.975–0.990)	< 0.001	1.000 (0.998–1.003)	0.768

*IRR* Incidence rate ratio, *CI* Confidence interval, *ART* Antiretroviral therapy. Treat-all: June 2016. PLHIV: People living with HIV. Region was divided into Northern China (Hohhot, Shijiazhuang, Beijing and Tianjin), Northeastern China (Dalian and Shenyang), Southern China (Guangzhou and Shenzhen), Southwestern China (Chongqing and Dehong), and Eastern China (Nanjing)

### Estimated time from infection to diagnosis

The median estimated time to diagnosis recorded before and after treat-all were 5.3 years (IQR: 3.0–8.2) and 6.1 years (IQR: 3.3–9.9), respectively. Poisson segmented regression analysis showed that the mean estimated time to diagnosis increased by 7.0% in the first month of treat-all (IRR = 1.070, 95% *CI*: 1.021–1.120; *P* = 0.004; Table 4 and Fig. 1C); mean estimated time to diagnosis showed an increase trend of 0.4% per month before treat-all (IRR = 1.004, 95% *CI*: 1.000–1.007; *P* = 0.044), and after treat-all showed an increasing trend of 0.1% per month (IRR = 1.001, 95% *CI*: 1.000–1.003; *P* = 0.330); by the end of 2019, mean estimated time to diagnosis was not significantly different from the expected level (IRR = 0.978, 95% *CI*: 0.811–1.178; *P* = 0.801). In the stratified analysis, mean estimated time to diagnosis in male (IRR = 1.056, 95% *CI*: 1.010–1.104; *P* = 0.016), female (IRR = 1.148, 95% *CI*: 1.062–1.240; *P* < 0.001), aged 26–35 (IRR = 1.053, 95% *CI*: 1.001–1.109; *P* = 0.048) and > 50 (IRR = 1.078, 95% *CI*: 1.000–1.161; *P* = 0.046), heterosexual transmission (IRR = 1.124, 95% *CI*: 1.042–1.213; *P* = 0.002) and Southwestern China (IRR = 1.129, 95% *CI*: 1.055–1.208; *P* < 0.001) showed significant increases in the first month of treat-all. By the end of 2019, mean estimated time to diagnosis in age > 50 (IRR = 0.737, 95% *CI*: 0.551–0.986; *P* = 0.045) and Eastern China (IRR = 0.289, 95% *CI*: 0.189–0.443; *P* < 0.001) was lower than expected levels, while it was higher than expected levels in Northeastern China (IRR = 1.484, 95% *CI*: 1.266–1.740; *P* < 0.001). The results were similar across route of transmission groups, and in a sensitivity analysis considering seasonality (Table 5 and Fig. 2C).

**Table 4:** Poisson segmented regression models of the impact of treat-all on mean estimated time from infection to diagnosis among PLHIV in China 1 January 2015 to 31 December 2019

	IRR (95% *CI*)							
	Level change at treat-all	*P* value	Level change in December 2019	*P* value	Trend before treat-all	*P* value	Trend after treat-all	*P* value
Mean estimated time to diagnosis	1.070 (1.021–1.120)	0.004	0.978 (0.811–1.178)	0.801	1.004 (1.000–1.007)	0.044	1.001 (1.000–1.003)	0.330
Gender								
Male	1.056 (1.010–1.104)	0.016	1.010 (0.846–1.206)	0.902	1.003 (1.000–1.007)	0.045	1.002 (1.001–1.003)	< 0.001
Female	1.148 (1.062–1.240)	< 0.001	0.847 (0.714–1.005)	0.065	1.005 (1.002–1.009)	0.001	0.998 (0.997–1.000)	0.094
Age, years								
0–25	1.044 (0.979–1.113)	0.185	1.121 (0.828–1.518)	0.452	0.999 (0.993–1.005)	0.730	1.001 (1.000–1.002)	0.049
26–35	1.053 (1.001–1.109)	0.048	1.137 (0.928–1.394)	0.208	1.000 (0.996–1.004)	0.968	1.002 (1.001–1.003)	0.011
36–50	1.057 (0.987–1.132)	0.116	0.858 (0.682–1.078)	0.182	1.006 (1.002–1.011)	0.003	1.001 (1.000–1.003)	0.214
> 50	1.078 (1.000–1.161)	0.046	0.737 (0.551–0.986)	0.045	1.009 (1.003–1.014)	0.001	1.000 (0.998–1.001)	0.604
Route of transmission								
Heterosexual	1.124 (1.042–1.213)	0.002	0.831 (0.584–1.181)	0.306	1.007 (1.000–1.013)	0.048	0.999 (0.998–1.001)	0.413
Homosexual	0.996 (0.940–1.055)	0.907	1.100 (0.967–1.251)	0.139	1.001 (0.998–1.003)	0.520	1.003 (1.002–1.005)	< 0.001
Others	1.014 (0.952–1.079)	0.705	1.054 (0.767–1.449)	0.775	1.000 (0.994–1.007)	0.875	1.001 (1.000–1.003)	0.331
Region								
Northern China	0.994 (0.961–1.029)	0.776	1.141 (0.996–1.306)	0.055	0.997 (0.995–1.000)	0.064	1.001 (0.999–1.002)	0.235
Northeastern China	0.962 (0.873–1.059)	0.440	1.484 (1.266–1.740)	< 0.001	0.995 (0.992–0.999)	0.013	1.006 (1.003–1.008)	< 0.001
Southern China	1.015 (0.938–1.098)	0.760	1.399 (0.917–2.133)	0.125	0.994 (0.986–1.003)	0.205	1.002 (1.001–1.003)	0.003
Southwestern China	1.129 (1.055–1.208)	< 0.001	0.772 (0.591–1.008)	0.057	1.009 (1.004–1.014)	0.001	1.000 (0.998–1.001)	0.781
Eastern China	0.877 (0.773–1.005)	0.063	0.289 (0.189–0.443)	< 0.001	1.026 (1.017–1.035)	< 0.001	0.999 (0.997–1.002)	0.616

*IRR* Incidence rate ratio, *CI* Confidence interval, *ART* Antiretroviral therapy. Treat-all: June 2016. PLHIV: People living with HIV. Region was divided into Northern China (Hohhot, Shijiazhuang, Beijing and Tianjin), Northeastern China (Dalian and Shenyang), Southern China (Guangzhou and Shenzhen), Southwestern China (Chongqing and Dehong), and Eastern China (Nanjing)

**Table 5:** Sensitivity analyses accounting for seasonality in the analysis of the impact of treat-all on three indicators of ART among PLHIV in China 1 January 2015 to 31 December 2019^#^

	IRR (95% *CI*)							
	Level change at treat-all	*P* value	Level change in December 2019	*P* value	Trend before treat-all	*P* value	Trend after treat-all	*P* value
Proportion of 30-day ART initiation*	1.116 (1.057–1.179)	< 0.001	1.006 (0.727–1.394)	0.969	1.011 (1.004–1.017)	0.002	1.008 (1.007–1.009)	< 0.001
Mean CD4^†^	0.996 (0.995–1.038)	0.844	1.002 (0.876–1.146)	0.979	0.997 (0.995–1.000)	0.056	0.997 (0.997–0.998)	< 0.001
Mean estimated time to diagnosis^†^	1.070 (1.024–1.118)	0.002	0.970 (0.856–1.099)	0.646	1.004 (1.001–1.006)	0.002	1.001 (1.000–1.003)	0.043

*IRR* Incidence rate ratio, *CI* Confidence interval, *ART* Antiretroviral therapy. Treat-all: June 2016. PLHIV: People living with HIV. #: three indicators include proportion of 30-day ART initiation, mean CD4 counts (cells/μL) at ART initiation, and mean estimated time from infection to diagnosis (year). *: using quasi-Poisson segmented regression models. †: using Poisson segmented regression models

## Discussion

In this study, we identified significant changes of the time from diagnosis to ART initiation and estimated time from infection to diagnosis after the implementation of treat-all policy in China. In the first month of treat-all, there was a significant increase in proportions of 30-day ART initiation (+ 12.6%) and mean estimated time to diagnosis (+ 7.0%). These suggested that the implementation of treat-all policy in China has achieved several positive results. However, mean CD4 at ART initiation did not appear to be significantly affected by treat-all in the first month but showed downward trend (-0.3% per month). Although the time from diagnosis to ART initiation increased significantly, expanded testing identified more late detection HIV cases with lower CD4 levels, which has led to a downward trend in mean CD4 in recent years. The sensitivity analysis suggested that the seasonal activity of the clinic did not appear to have an impact on the results.

We found that the median time from diagnosis to ART initiation before and after treat-all in China was 41 days (IQR: 18–256) and 22 days (IQR: 11–65), respectively, which aligns with previous studies. A retrospective nationwide cohort study conducted in China between 1 January 2012 and 30 June 2014 [28] found a median time from diagnosis to CD4 test of 6 days (IQR: 0–20) and a median time from CD4 to ART of 10 days (IQR: 4–18) in those who started ART within 30 days; and 11 days (IQR: 1–39) and 197 days (IQR: 109–283) in those who started ART > 30 days, respectively. Similarly, a longitudinal cohort study of gay and bisexual men in Australia[29] reported a median time from HIV diagnosis to linkage to care of 3 days (IQR: 0–4) and a median time from linkage to care to ART initiation of 21 days (IQR: 21–25) in 2016–2017; and 0 days (IQR: 0–3) and 15 days (IQR: 14–18) in 2018–2019, respectively. Another study in Italy [30] found that median days from HIV diagnosis to start of combination ART was 40 (IQR: 21–73) in 2016–2017.

Regarding the proportion of 30-day ART initiation, our study observed an immediate increase of 12.6% in the first month of treat-all in China, which is consistent with existing studies. A regression discontinuity analysis conducted in 6 sub-Saharan African countries [14] found that after country-level adoption of the treat-all policy, the proportion of ART initiation within 30 days of enrolment in HIV care significantly increased in four countries, ranging from 12.5% to 34.5%. Another study in ten Rwandan health centers [10] found there was a 31.3% increase in the predicted probability of 30‐day ART initiation (95% *CI:* 15.5–47.2) immediately after treat-all implementation, with a subsequent increase of 1.1% per month (95% *CI:* 0.1–2.1); At the end of the study period, 30‐day ART initiation was 95.2%, or 47.8% (95% *CI:* 8.1–87.8) above what would have been expected under the pre‐treat-all trend. However, the proportion of 30-day ART initiation in China was only 65.6% by December 2019, much lower than the 95.2% in Rwanda in September 2017 [10], and other African countries [14]. This may be due to the fact that “rapid initiation” policy in China was implemented later than the treat-all policy. The World Health Organization introduced the concept of "rapid initiation" in 2017, recommending that PLHIV initiate ART within 7 days of diagnosis, and patients who are ready for treatment and have no clinical contraindications or other special circumstances initiate ART on the day of diagnosis [31]. When the South African Government changed the policy to universal test and treat, most clinics started same-day ART initiation for PLHIV [32]. However, China did not incorporate the concept of "rapid initiation" into its Guidelines for Diagnosis and Treatment of HIV/AIDS until 2021 [33]. In addition, there are significant regional differences in China in terms of HIV epidemic, policies, level of economic development, and medical care, which may lead to a prolonged period between policy implementation and full execution.

Although our study did not identify a significant change in the proportion of 30-day ART initiation by gender and age in the first month of treat-all policy, we found that females showed a 4.8% (IRR = 1.048, 95% *CI:* 0.831–1.321) increase and males showed a 3.1% (IRR = 0.969, 95% *CI:* 0.859–1.095) decrease. Furthermore, females were found to show a significant increase trend after treat-all, while males not significant. The dominant discourse that men are strong and healthy visibly makes it difficult to accept the fact that they are HIV-positive, which poses a challenge for them to seek timely treatment [34]. Previous studies suggested that ART initiation in men and young adults should receive more attention. For instance, a study from six clinics in Johannesburg, South Africa [8] showed women had higher 30-day ART rates compared with men (a*HR* = 1.2, 95% *CI:* 1.0–1.4). Another study from Rwanda [10] found ART initiation within 30 days was less likely among patients aged 15 to 24 compared to > 24 years (a*RR* = 0.93, 95% *CI:* 0.87–1.00). Data from rural South Africa [7] found women consistently initiated ART at higher CD4 counts than men before and during treat-all. Other studies have reported lower rates of ART initiation in men and young adults [35, 36], and young people were less likely to be virally suppressed [37]. In addition, the high proportion of male participants in our data is consistent with the actual situation in China [23, 38].

The overall decrease in mean CD4 at ART initiation was negligible (-1.0%, not significant) in the first month of treat-all in China. However, data from 17 public sector primary care clinics in rural South Africa [7] showed that mean CD4 at ART initiation increased from 317.1 cells/μl (95% *CI*: 308.6–325.6) 1 to 8 months prior to treat-all to 421.0 cells/μl (95% *CI*: 413.0–429.0) 1 to 12 months after treat-all, then subsequently fell to 389.5 cells/μl (95% *CI*: 381.8–397.1) 13 to 30 months after treat-all. Although the South African study also found a transient downward trend in mean CD4 one year after treat-all implementation (-5.6 cells/µl, 95% *CI:* -7.5–-3.8), the trend became flat after one year (+ 1.0 cells/µl, 95% *CI:* -0.3–2.2). In addition, the median of CD4 in China was lower than most countries (before treat-all: 277 cells/µl, IQR: 158–389; after treat-all: 235 cells/µl, IQR: 98–370). This may be due to China's primary focus on implementing a universal testing policy. In our study, we found that there was a 7.0% significant increase in mean estimated time to diagnosis in the first month of treat-all. After treat-all, PLHIVs were significant older (median: 40 years old, IQR: 28–54) at ART initiation than before (median: 36 years old, IQR: 27–49). These findings, along with the mean CD4 results, reflect the impact of expanded HIV testing in China.

The reason why the impact of treat-all policy was mainly seen in HIV testing in China may be due to the uneven distribution of medical resources and the principle of voluntary treatment. The number of hospital beds and doctors distribute unevenly among provinces in China, with relatively less reserves per capita in Yunnan, Guangdong and Guangxi [39]. Given the high prevalence of HIV/AIDS in these regions, the difficulty to start ART rapidly was exacerbated by medical inequalities [40]. Yunnan, one of the provinces worst-hit by the HIV epidemic in the country, reported slow progress in treat-all policy implementation. The main obstacles are the difficulty in disseminating knowledge about HIV, the shortage of HIV allied health professionals, the uneven distribution of medical resources, and the stigmatization of PLHIV [41]. In addition, although the criterion of CD4 + T-cell count used to determine whether to initiate ART was removed, due to the adoption of the voluntary principle, people with high CD4 + T-cell counts may consider themselves to be in good health and did not need to initiate ART immediately [42]. There are many other factors that can affect their ART initiation compliance, such as social stigma, unable to disclose, etc. [43]. And those with low CD4 + T-cell counts may want to initiate ART as soon as possible because they are worried about disease progression, thus leading to the results presented in this study. Based on such a hypothesis, services including health education, social and emotional support and ART support, after HIV diagnosis, should be in place to ensure early ART initiation.

In regions characterized by a high per capita reserve of hospital beds, as exemplified by Northern China and Northeastern China [39], immediate increase in mean CD4 after treat-all implementation (+ 3.3% and + 8.0%, respectively) suggesting that these regions may have effectively implemented rapid ART policy. However, PLHIV in China are required to undergo a sequence of laboratory tests, including HIV antibody screening tests, repeat tests, antibody confirmatory tests, nucleic acid tests, and CD4 T-cell tests before initiating ART, which can extend over several weeks. This, along with the prevailing stigma surrounding PLHIV in Chinese society, may contribute to attrition rates in treatment. A 15-year study in Yunnan[44] found an overall rate of 6.77/100 person-years (95% *CI*: 6.69–6.85) of attrition among 110,373 PLHIV receiving ART from 2004–2018. It is worth noting that full implementation of the policy in the short term may be challenging due to China’s vast geographical size and the uneven distribution of the HIV epidemic and health care resources. More research is needed to comprehend the specific difficulties within different regions and devise contextualized solutions. Stakeholders such as governmental bodies, medical institutions, and social agencies must allocate more attention, enhance cooperation, and learn from successful experiences abroad to explore strategies suitable for China.

Our study has several limitations. Firstly, it is an ecological study and cannot demonstrate a causal relationship between treat-all policy and changes in time from diagnosis to ART initiation and CD4 + T cell counts at ART initiation in China. Our results should be interpreted as a correlation between the implementation of the treat-all policy and these outcomes. Secondly, we were unable to further examine the impact on viral load even though it is an important indicator. Due to limited resources in most regions, it was not feasible to perform HIV RNA load testing on patients as required. Thirdly, given the COVID-19 has significant impact on the HIV care continuum [23], we did not study data after 2020. Studies of subsequent changes and effects on rapid ART initiation should control for the impact of COVID-19-related interruptions. Finally, it is important to note that the limited representation of certain regions in our study may limit the generalizability of our findings. If more data become available in the future, it would be beneficial to conduct studies with more representative samples. Our study was based on long term observation of a large population covering a variety of regions in China, which ensured the representativeness of the sample and the robustness of the findings. We adjusted for long-term trends to elucidate the impact of treat-all on time from diagnosis to ART initiation, CD4 + T cell counts at ART initiation, and mean estimated time to diagnosis in different characteristics of PLHIV in China. Difficulties of rapid ART in China may require additional efforts to address.

## Conclusions

The implementation of treat-all policy in China was associated with a positive effect on HIV care and treatment outcomes. Our study points out the unbalanced rapid ART implementations in different regions in China. Subsequent studies on the time from diagnosis to ART initiation and CD4 + T cell counts at ART initiation are needed to provide a clearer picture of the longer impact of treat-all.

### Supplementary Information

**Additional file 1: Table S1.** Estimates of the random intercept and random slope of the CD4+ T-cell elimination model, based on the CD4 elimination model parameters estimated from HIV/AIDS data in the National AIDS Case Report Database in China [2].

## Data Availability

The processed data that support the findings of this study are available on request from the corresponding author, HZ. The raw data are not publicly available due to their containing information that could compromise the privacy of research participants.

## Abbreviations

ART: Antiretroviral therapy
PLHIV: People living with HIV
IQR: Interquartile range
IRR: Incidence rate ratio
UTT: Universal test and treat
MSM: Men who have sex with men
NFATP: National Free Antiretroviral Treatment Program
CDC: Center for Disease Control and Prevention
*HR*: Hazard ratio
*RR*: Risk ratio
*CI*: Confidence interval
