# Supplementary material for: Immediate and long-term outcomes after treat-all among people living with HIV in China: an interrupted time series analysis
Source: Infect Dis Poverty. 2023 Aug 14;12:73. doi: 10.1186/s40249-023-01119-7 (PMC10424386; doi:10.1186/s40249-023-01119-7)
Supplement: Supplementary file 1 — Additional file 1: Table S1. Estimates of the random intercept and random slope of the CD4+ T-cell elimination model, based on the CD4 elimination model parameters estimated from HIV/AIDS data in the National AIDS Case Report Database in China [2]. [file 40249_2023_1119_MOESM1_ESM.pdf]

## Appendix

### Description of the back-calculation method

Previous studies have demonstrated, through laboratory-based research, that the levels of CD4 and viral load in HIV-infected individuals exhibit an inverse relationship over time, revealing the natural disease progression from HIV infection to clinical symptoms [1]. However, due to delayed diagnosis in most HIV-infected individuals, CD4 changes after HIV infection cannot be observed immediately. Therefore, based on the decline of CD4 over time in HIV-infected individuals, we used the square root of CD4 to fit the relationship between CD4 and time, constructing a CD4 elimination model to estimate the time of HIV infection of PLHIV.

### Formula for back-calculation method based on the CD4 elimination model parameters

*Linear elimination relationship between the square root of CD4 and the duration of infection based on a linear mixed effects model fitted to the pattern of CD4 decline over time prior to ART initiation*

$$\sqrt{CD_4(T_i)} = a_i + (b_i \times T_i) + e_{it}$$

Where $T_i$ is the time interval between the date of HIV infection and the first CD4 test, $e_{it}$ is the random error.

*Calculating the estimated time from infection to diagnosis, based on CD4 elimination model fitted using HIV/AIDS data in the National AIDS Case Report Database in China*

$$T_i = \frac{\sqrt{firstCD_4} - a_i}{b_i}$$

**Additional file 1 Table S1 Estimates of the random intercept and random slope of the CD4+ T-cell elimination model, based on the CD4 elimination model parameters estimated from HIV/AIDS data in the National AIDS Case Report Database in China [2]**

| Gender | Age group | Route of transmission | Estimated value of intercept (95% CI) | Estimated value of slope (95% CI) |
|---|---|---|---|---|
| Male | 15-24 | Homosexual | 24.84(23.76, 25.92) | -1.31(-1.33, -1.25) |
| | 25-34 | | 23.94(22.86, 25.02) | -1.37(-1.40, -1.33) |
| | 35-44 | | 23.44(21.91, 24.96) | -1.53(-1.58, -1.47) |
| | >45 | | | -1.59(-1.68, -1.51) |
| Male | 15-29 | Heterosexual | 24.42(22.64, 26.20) | -1.21(-1.24, -1.18) |
| | 30-39 | | 23.17(21.25, 25.09) | -1.27(-1.30, -1.24) |
| | >40 | | 24.04(21.28, 26.80) | -1.48(-1.51, -1.44) |
| Female | 15-29 | | 23.80(21.49, 26.12) | -1.22(-1.25, -1.19) |
| | 30-39 | | 22.55(17.73, 27.37) | -1.27(-1.31, -1.23) |
| | >40 | | 22.62(20.37, 24.88) | -1.46(-1.50, -1.41) |
| Male | 15-29 | Others | -1.18(-1.24, -1.12) | -0.88(-0.91, -0.84) |
| | 30-39 | | -1.12(-1.17, -1.07) | -0.93(-0.97, -0.90) |
| | >40 | | -1.06(-1.14, -0.98) | -1.00(-1.05, -0.94) |
| Female | 15-29 | | -1.17(-1.30, -1.03) | -0.98(-1.06, -0.90) |
| | 30-39 | | -1.05(-1.17, -0.93) | -1.04(-1.13, -0.95) |
| | >40 | | -0.87(-1.04, -0.69) | -1.17(-1.34, -1.00) |

**Formula for Poisson and quasi-Poisson segmented regression models**

*Calculating the change in outcome at "treat-all", unadjusted for seasonality*

$$\log \mathrm{Y_t} = b_0 + b_1(t - T) + b_2x + b_3x(t - T)$$

Where $\mathrm{Y_t}$ is the outcome variable at time $t$, $t$ represents the elapsed time in months since the start of the study, $x$ is a dummy variable indicating the implementation of "treat-all" policy, and $T$ is the center time.

*Calculating the change in outcome at "treat-all", adjusted for seasonality*

$$\log \mathrm{Y_t} = b_0 + b_1(t - T) + b_2x + b_3x(t - T) + b_4 \sin(\frac{2\pi t}{12}) + b_5 \cos(\frac{2\pi t}{12}) + b_6 \sin(\frac{4\pi t}{12}) + b_7 \cos(\frac{4\pi t}{12})$$

Where $\sin(\frac{2\pi t}{12}), \cos(\frac{2\pi t}{12})$, $\sin(\frac{4\pi t}{12}), \cos(\frac{4\pi t}{12})$ are two sine and cosine pairs of Fourier terms used to adjust the seasonality.

*Counterfactual model, unadjusted for seasonality*

$$\log \mathrm{Y_t} = b_0 + b_1(t - T)$$

*Counterfactual model, adjusted for seasonality*

$$\log \mathrm{Y_t} = b_0 + b_1(t - T) + b_4 \sin(\frac{2\pi t}{12}) + b_5 \cos(\frac{2\pi t}{12}) + b_6 \sin(\frac{4\pi t}{12}) + b_7 \cos(\frac{4\pi t}{12})$$

**Sample R code for Poisson and quasi-Poisson regression models, using proportions of 30-day ART initiation as an example**

```
# Load packages
library(tsModel)
library(tidyverse)
library(sandwich)
library(dplyr)
library(qcc)

# Centre time
list2$time_c <- list2$n - 18 # Time centered at the first month of “treat-all”
list2$time_e <- list2$n - 60 # Time centered at the end of 2019

# Overdispersion test
qcc.overdispersion.test(list2$x)

# Quasi-Poisson regression model of proportions of 30-day ART initiation without adjusting for seasonality
```

```
model_time <- glm(x ~ interruption + n + interruption:time_c, family = quasipoisson, list2)
summary(model_time)

# Calculated the impact of “treat-all” at the end of 2019 without adjusting for seasonality
model_time_end <- glm(x ~ interruption + n + interruption:time_e, family = quasipoisson, list2)
summary(model_time_end)

# Look at residuals for autocorrelation
acf(residuals(model_time, type = "deviance"))
pacf(residuals(model_time, type = "deviance"))
acf(residuals(model_time_end, type = "deviance"))
pacf(residuals(model_time_end, type = "deviance"))

# Calculate Newey-West standard errors, with the lag taking the optimal value calculated
est <- exp(c(coef(model_time)["interruption:time_c"], coef(model_time)["interruption"], coef(model_time)["n"]))
se1 <- sqrt(diag(NeweyWest(model_time, prewhite = F)))["interruption:time_c"]
se2 <- sqrt(diag(NeweyWest(model_time, prewhite = F)))["interruption"]
```

```
se3 <- sqrt(diag(NeweyWest(model_time, prewhite = F)))["n"]

lb <- est * exp(-1.96 * c(se1, se2, se3))

ub <- est * exp(1.96 * c(se1, se2, se3))

table <- cbind(round(est, digits = 3), round(lb, digits = 3), round(ub, digits = 3))

table


# Calculate trend and confidence intervals after “treat-all”

V <- NeweyWest(model_time, prewhite = F)

se <- sqrt(V["n", "n"] + V["interruption:time_c", "interruption:time_c"]+ 2 * V["n", "interruption:time_c"])

slope_post <- sum(coef(model_time)[c("n", "interruption:time_c")])

lower <- slope_post - 1.96 * se

upper <- slope_post + 1.96 * se

round(exp(cbind(slope_post, lower, upper)),3)


# Quasi-Poisson regression model of proportions of 30-day ART initiation adjusting for seasonality

model_time_sea <- glm(x ~ interruption + n + interruption:time_c +harmonic(month, 2, 12), family = quasipoisson, list2)

summary(model_time_sea)
```

**References**


1. Fauci, A.S., et al., Immunopathogenic mechanisms of HIV infection. Annals of Internal Medicine, 1996. 124(7): p. 654-663.

2. Tang, L., Estimating the trend of new HIV infections in Honghe Prefecture, Yunnan Province based on the back-calculation method. 2020, Chinese Centre for Disease Control and Prevention.
